# Surface physical cues mediate the uptake of foreign particles by cancer cells

**DOI:** 10.1063/5.0138245

**Published:** 2023-03-20

**Authors:** Katerina Tischenko, Yifat Brill-Karniely, Eliana Steinberg, Hadas Segev-Yekutiel, Ofra Benny

**Affiliations:** 1 Institute for Drug Research, The School of Pharmacy, Faculty of Medicine, The Hebrew University of Jerusalem, Jerusalem 9112001 Israel; 2 The Core Research Facility, Faculty of Medicine, The Hebrew University of Jerusalem, Jerusalem 9112001 Israel

## Abstract

Cancer phenotypes are often associated with changes in the mechanical states of cells and their microenvironments. Numerous studies have established correlations between cancer cell malignancy and cell deformability at the single-cell level. The mechanical deformation of cells is required for the internalization of large colloidal particles. Compared to normal epithelial cells, cancer cells show higher capacities to distort their shapes during the engulfment of external particles, thus performing phagocytic-like processes more efficiently. This link between cell deformability and particle uptake suggests that the cell's adherence state may affect this particle uptake, as cells become stiffer when plated on a more rigid substrate and vice versa. Based on this, we hypothesized that cancer cells of the same origin, which are subjected to external mechanical cues through attachment to surfaces with varying rigidities, may express different capacities to uptake foreign particles. The effects of substrate rigidity on cancer cell uptake of inert particles (0.8 and 2.4 *μ*m) were examined using surfaces with physiologically relevant rigidities (from 0.5 to 64 kPa). Our data demonstrate a wave-like (“meandering”) dependence of cell uptake on the rigidity of the culture substrate explained by a superposition of opposing physical and biological effects. The uptake patterns were inversely correlated with the expression of phosphorylated paxillin, indicating that the initial passive particle absorbance is the primary limiting step toward complete uptake. Overall, our findings may provide a foundation for mechanical rationalization of particle uptake design.

## INTRODUCTION

Cancer is the leading cause of death worldwide, accounting for nearly 10 × 10^6^ deaths in 2020.^1^ Progression to the metastatic stage is one of the primary causes for this.^2,3^ Cancer cells differ from normal cells in their genetic, molecular, and morphological features, as well as their biomechanical properties. At the single-cell level, the mechanical deformability of cancer cells (ability to change shape) was found to correlate with malignancy potential and cell function.^4–11^ It is well established, for example, that mechanical forces in the tumor niche affect various biological functions of cancer cells, such as their proliferation and level of differentiation.^12,13^

Phagocytosis is a natural mechanism performed by immune cells that act as professional phagocytes such as macrophages and neutrophils and is largely employed as a defense mechanism against microbes.^14,15^ In these cells, phagocytosis is mediated by receptor–ligand interactions as well as the mechanical parameters of the phagocytic target.^16^ In contrast, for nonprofessional phagocytes such as fibroblasts, epithelial cells, and cancer cells, no specific receptors involved in the engulfment of microparticles have been identified.^17^ Therefore, for these cases, the term phagocytosis is used to designate any cellular internalization of particles that are larger than the endocytosis particle-size threshold and is independent of biological mediators. Small nanoparticles (<300 nm) are often taken up by cells via endocytosis, a process involving a variety of biological mediators including clathrin and caveolin.^18–20^ Mechanically, endocytosis is predominantly governed by membrane fluidity and local distortions rather than massive deformations of the cell body.^21^ However, in the case of phagocytosis, which is the dominant uptake pathway for particles larger than 500 nm, the biomechanical mechanisms primarily involve massive and global cell distortions and cytoskeletal remodeling. This means that in these cases, the engulfment process is affected more by cell stiffness than by membrane fluidity. Our recent work demonstrated a Triangular Correlation (TrC) at the single-cell level between cancer cells' deformability, the extent of phagocytosis, and malignancy.^22^ This key finding implies that cells that are more deformable have a greater capacity to engulf large particles, but it also suggests that the temporal mechanical state of cells may affect this capacity as well.

The mechanical state of cell results from the combined effects of native structural effectors such as the cytoskeleton, membrane, water content, and adaptive effectors that are resulting from external mechanical inputs. Given the relationship between cell mechanics and phagocytosis, we hypothesize that the cell's capacity to engulf colloidal particles (sub-micron and micrometer particles) may be affected by cells' adherence to surfaces. Despite the significant biological implications of this relationship, particularly in relation to cancer cell phagocytosis of synthetic or natural particles, this aspect of cell uptake behavior has not been thoroughly researched. While the extent and nature of cell adhesion to surfaces may be affected by many surface properties, such as texture, composition, and rigidity, our current study focuses specifically on the effect of surface rigidity.

The rigidity of the surface to which cells adhere affects both the physical properties of cells, such as spreading area, stretching, and Young's modulus,^19–22^ as well as their biological activities, including proliferation, motility, morphology, differentiation, and adhesion.^25^ Therefore, mechanical-mediated phagocytosis may occur in a variety of clinical scenarios. Examples include cancer metastases, in which cancer cells are attached to secondary organs, each with distinct rigidity, or when cells are located on the outer lining of a tissue that becomes stiffer over time due to fibrosis or desmoplasia.

Here, we investigated the adhesion of cancer cells to surfaces of distinct rigidities in pancreatic ductal adenocarcinoma (PDAC) and breast adenocarcinoma cell lines, both of which are known for their adaptability to complex mechanical desmoplastic niches *in vivo*.^23,24^ These tumors are distinguished by abnormal tissue stiffening, which affects the cellular phenotypes of cancer and stromal cells in tumors as well as cancer progression in general.

Detailed uptake experiments were performed on gel surfaces with distinct rigidities mimicking natural tissues. Fabricated polyacrylamide hydrogels and commercial silicone gels provided the necessary physiologically relevant range of Young's modulus values, thereby rendering a well-controlled mechanically mediated platform. The uptake experiments were performed with inert fluorescently labeled sub-micron and micrometer particles (0.8 and 2.4 *μ*m) that were incubated with the cells for different durations and taken up via a phagocytic-like mechanism, as previously shown.^22^

Using imaging flow cytometry, which allows cell imaging and particle localization analysis, we quantified the efficiency of particle uptake within the cells. Our data revealed that the cells' uptake pattern showed a wave-like (meandering) dependence on the rigidity of the substrate on which they were cultured. This meandering behavior indicates the presence of opposing physical and biological factors that control the cells' uptake capacity, as shown experimentally and theoretically.

There are two phases in ligand-mediated phagocytosis as performed by professional phagocytes. The first is a contact phase that begins slowly, possibly due to the passive cell–particle interaction, while the second is an active stage, during which particle engulfment rapidly develops.^25^ Therefore, one of our goals in the present work was to determine whether the two phases that occur in phagocytosis, i.e., the passive and active phases, are also present during the uptake of bare particles by cancer cells. To elucidate this, we measured the expression of phosphorylated paxillin in cells grown on surfaces of various rigidities. Since phosphorylated paxillin is expected to be negatively correlated with passive absorbance and positively correlated with active engulfment, its expression levels can be used to determine which of these two processes is primarily responsible for determining the extent of uptake. Interestingly, we found that the expression of phosphorylated paxillin was inversely correlated with microparticle uptake, indicating that the first stage, at which passive adhesion occurs, is the main limiting step.

**FIG. 1.**
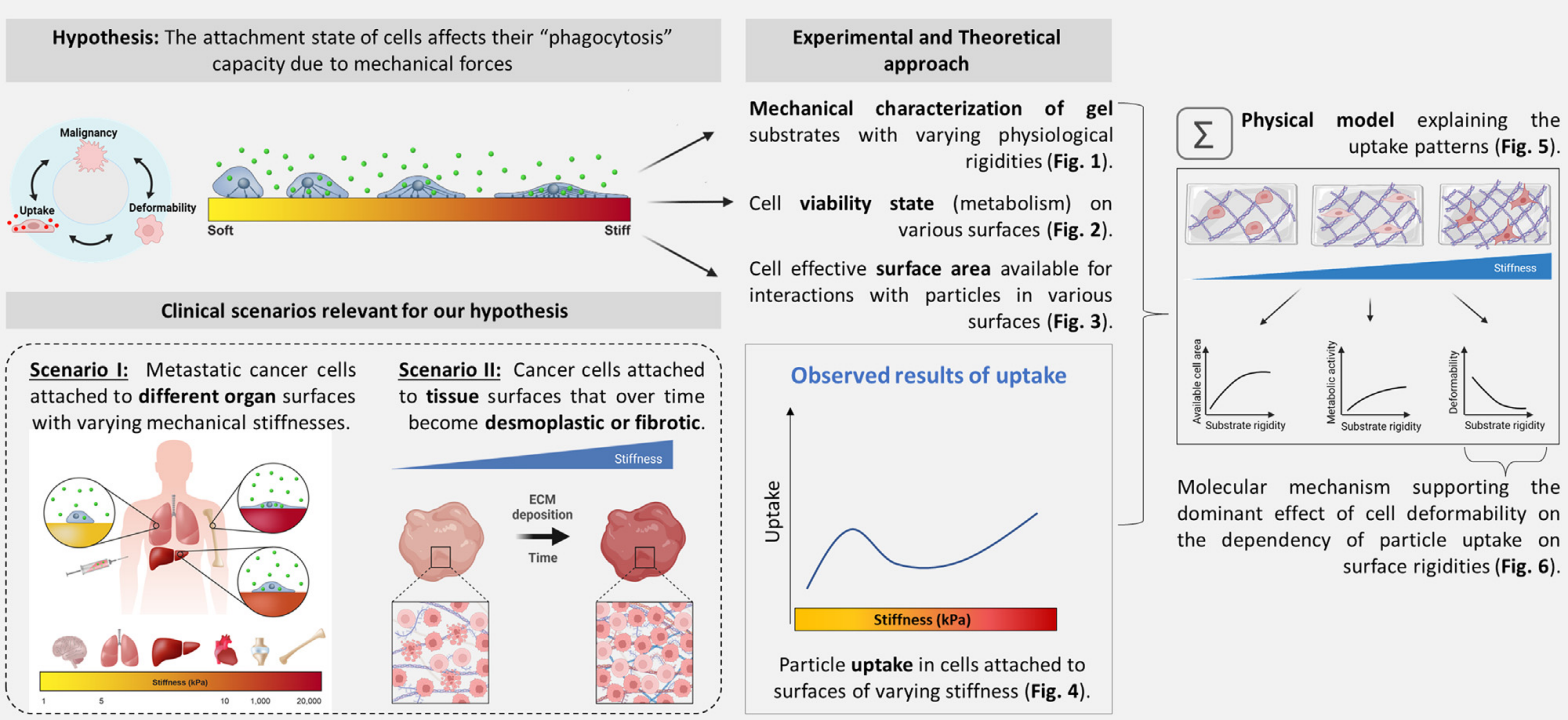
The hypothesis and outline of our study investigating how the influence of external stimuli on the mechanical states of cells can modify the cell's capacity to uptake particles via deformation-mediated processes. Two major biological scenarios are relevant to our hypothesis: (1) cells being subjected to static external mechanical stimuli, such as in the early tissue adherence of metastatic cells; and (2) cells being subjected to dynamic external mechanical stimuli, such as in cancer cells attached to desmoplastic tissue that is stiffening over time, which commonly occurs during tumor maturation and progression. There are opposing trends that may control the dependence of the cellular uptake level on increasing surface rigidity: the increase in cell spreading area, which increases the number of particles in contact with each cell; the elevation in cell stiffness, which increases the energetic penalty associated with the cell deformation required for particle adhesion, and the elevated cell metabolism that indicate cell viability on more rigid substrates. The non-monotonic dependence of particle uptake on the substrate rigidity, which is predicted from the physical arguments, reflects an initial increase followed by a decrease in uptake when increasing substrate rigidity.

Taken together, our findings indicate that the tissues' mechanical traits can significantly influence the phagocytic behavior of adherent cancer cells. This suggests that cancer cell localization in the body, for example, in primary and metastatic organs, may significantly affect cell function, as indicated by the mechanically mediated uptake of particles. Further studies could explore the effect of various external mechanical cues on the cellular mechanical uptake of natural microparticles or synthetic microcarriers such as microbubbles, which are used for imaging or theranostics. Our findings may be further developed as a tool for the selective delivery of microparticles to specific organs in the body, such as tumor tissues. Moreover, based on the stiffness-dependent cellular interactions with drug carriers seen in physical models that account for 3D, additional physical parameters may be identified and used to improve targeted drug delivery systems and their selective uptake, substantially reducing the off-target drug exposure and increasing the success rate of cancer treatments. Figure 1 illustrates the general hypothesis and experimental outline to identify the mechanical effectors that are involved in phagocytosis in cells attached to various surfaces.

## RESULTS

### Measurement of polyacrylamide gel stiffness using a MicroTester

**FIG. 2.**
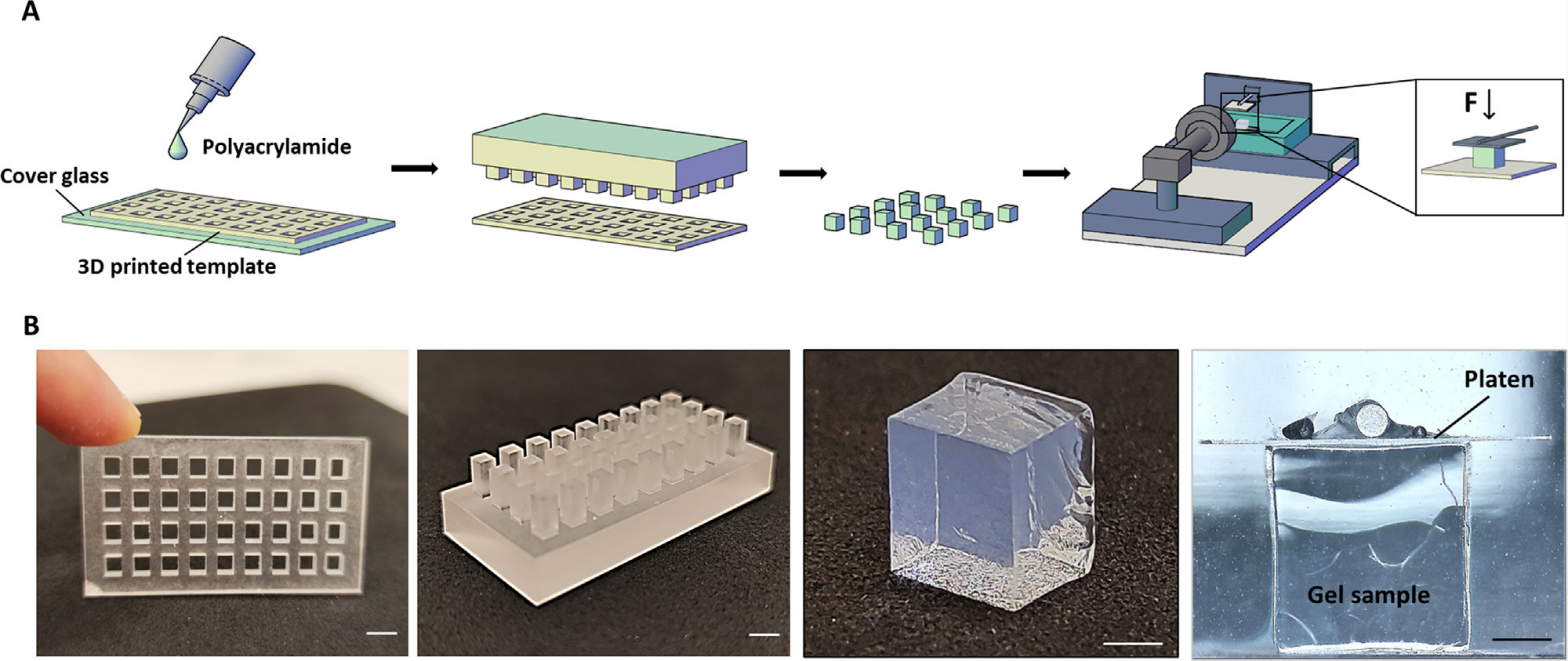
The polyacrylamide gel stiffness measurement procedure using the MicroTester. Polyacrylamide gels were fabricated using 3D-printed casting molds with appropriate dimensions for the MicroTester. (a) Illustration of the fabrication of gels and their transfer to the MicroTester. Polyacrylamide was cast into 3D-printed templates placed on cover glasses. After polymerization, complementary molds were used to remove them. Then gels were placed in the MicroTester and compressed with the tungsten platen. (b) Representative images of 3D-printed molds and gels. The 3D molds were printed using the Asiga Max-X27 UV printer. See also Fig. S1.

Hydrogels serve as a valuable and accessible culture model for extracellular matrices. Figure 2 shows the mechanical characterization of fabricated polyacrylamide (PAA) hydrogels via compressive experiments. The elastic modulus of the hydrogel was calculated from the applied stress and the resultant strain of the material within the linear elastic region of deformation.^29^ The gels were custom-fabricated using 3D-printed molds with dimensions compatible with the MicroTester scale analyzer. Polyacrylamide was cast into the molds and placed on cover glasses. After polymerization, complementary molds were used to remove the polyacrylamide gel cubes. Cubic samples of 1 mm^3^ were compressed with a tungsten compression platen attached to the edge of a microbeam (see Methods). The mean Young's moduli of polyacrylamide cubes were 1.59 ± 0.37, 9.19 ± 1.77, and 31.34 ± 0.50 kPa. Our elasticity results are generally consistent with previously reported studies, while minor discrepancies may be attributable to the selected measurement techniques.^30,31^ Despite the fact that the percentages of acrylamide and bisarylamide used in our studies were almost identical to the concentrations used in other studies, it is important to note that Young's modulus studies in the literature were conducted with cantilever-based micro-indentation mechanical testing, while our measurements were conducted using macroscale compression testing on bulk gel samples. However, there is generally good consistency between micromechanical measurements and bulk measurements, as shown in the study by Kain *et al.,*^32^ where the microscale mechanics of agarose samples measured using Atomic Force Microscopy showed good correlation with bulk compression tests.

### Substrate stiffness affects AsPC-1 cell metabolic activity

**FIG. 3.**
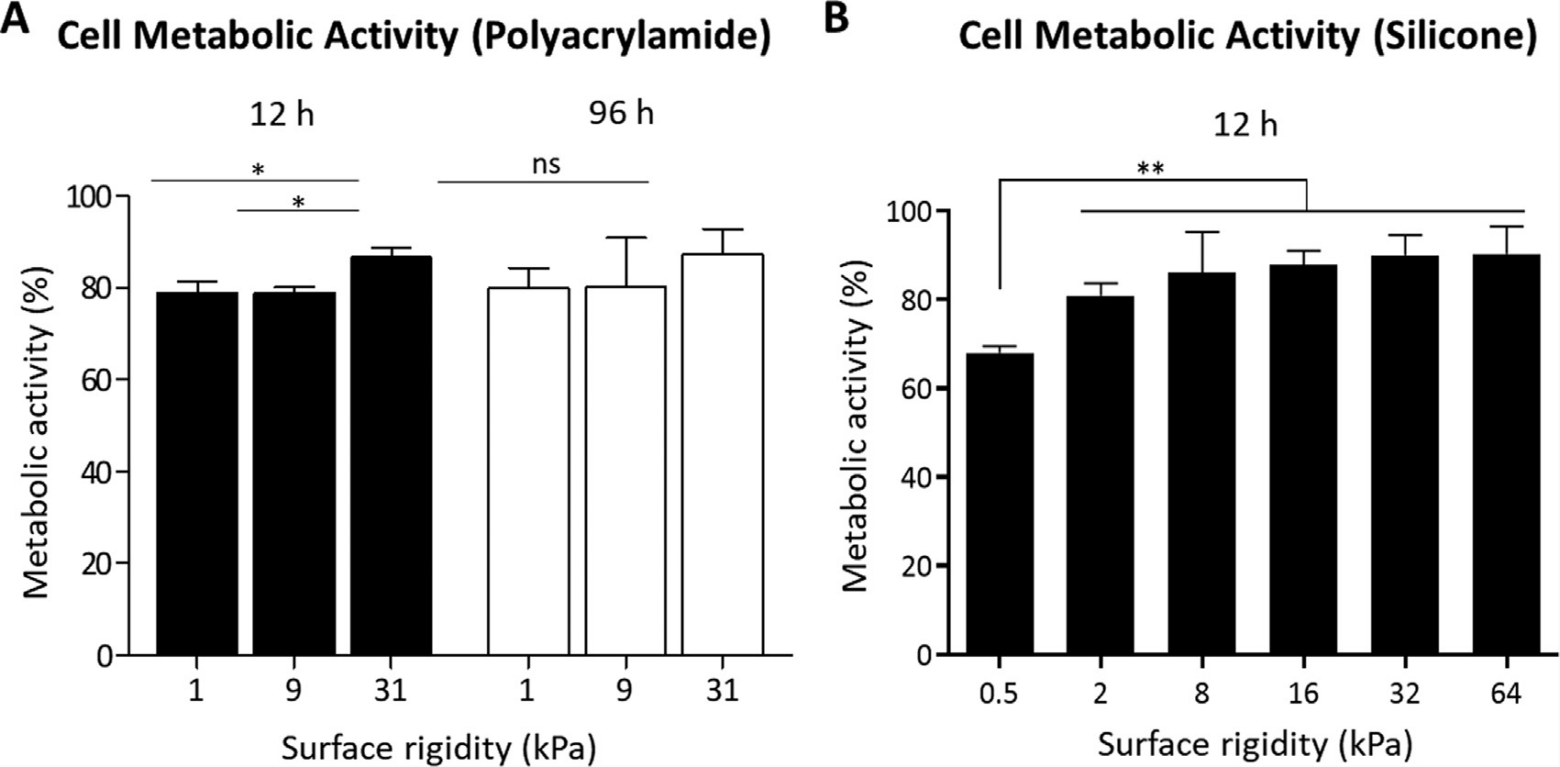
Substrate stiffness affects AsPC-1 cell metabolic activity. (a) Metabolic activity of AsPC-1 cells was measured via an MTT assay after 12 and 96 h of incubation on fabricated surfaces ranging in stiffness (1, 9, and 31 kPa). n = 6. ^*^p < 0.05. (B) The metabolic activity of AsPC-1 cells grown for 12 h on commercial surfaces of different rigidities was evaluated using an MTT assay. n = 6. ^**^p < 0.01.

Matrix stiffness regulates cell behavior in various ways. Cell metabolism reflects the cells' viability on different surfaces and may modify the cell's capacity to engulf particles. To evaluate the relationship between matrix stiffness and the cells' metabolic activity, AsPC-1 cells were grown on polyacrylamide-fabricated matrices of different rigidities for 12 or 96 h, followed by a 3–(4,5-dimethylthiazol-2-yl)-2,5-diphenyltetrazolium bromide (MTT) colorimetric assay [Fig. 3(a)]. In addition, the metabolic activity of cells grown on commercial substrates for 12 h was measured [Fig. 3(b)].

We found a maximum decrease in 30% in metabolic activity, which was observed with the soft substrates [Fig. 3(b)]. Thus, most cells maintained high functionality on fabricated polyacrylamide [Fig. 3(a)] as well as on the commercial [Fig. 3(b)] substrates tested.

To further assess the link between surface rigidity and cell death, we applied Annexin V–APC and PI double staining to AsPC-1 cells grown for 12 h on silicone surfaces of various stiffnesses. Figure S2 shows that more than 90% of the cells remained viable after 12 h of growth on different surfaces.

### Higher cell spreading on stiff surfaces

**FIG. 4.**
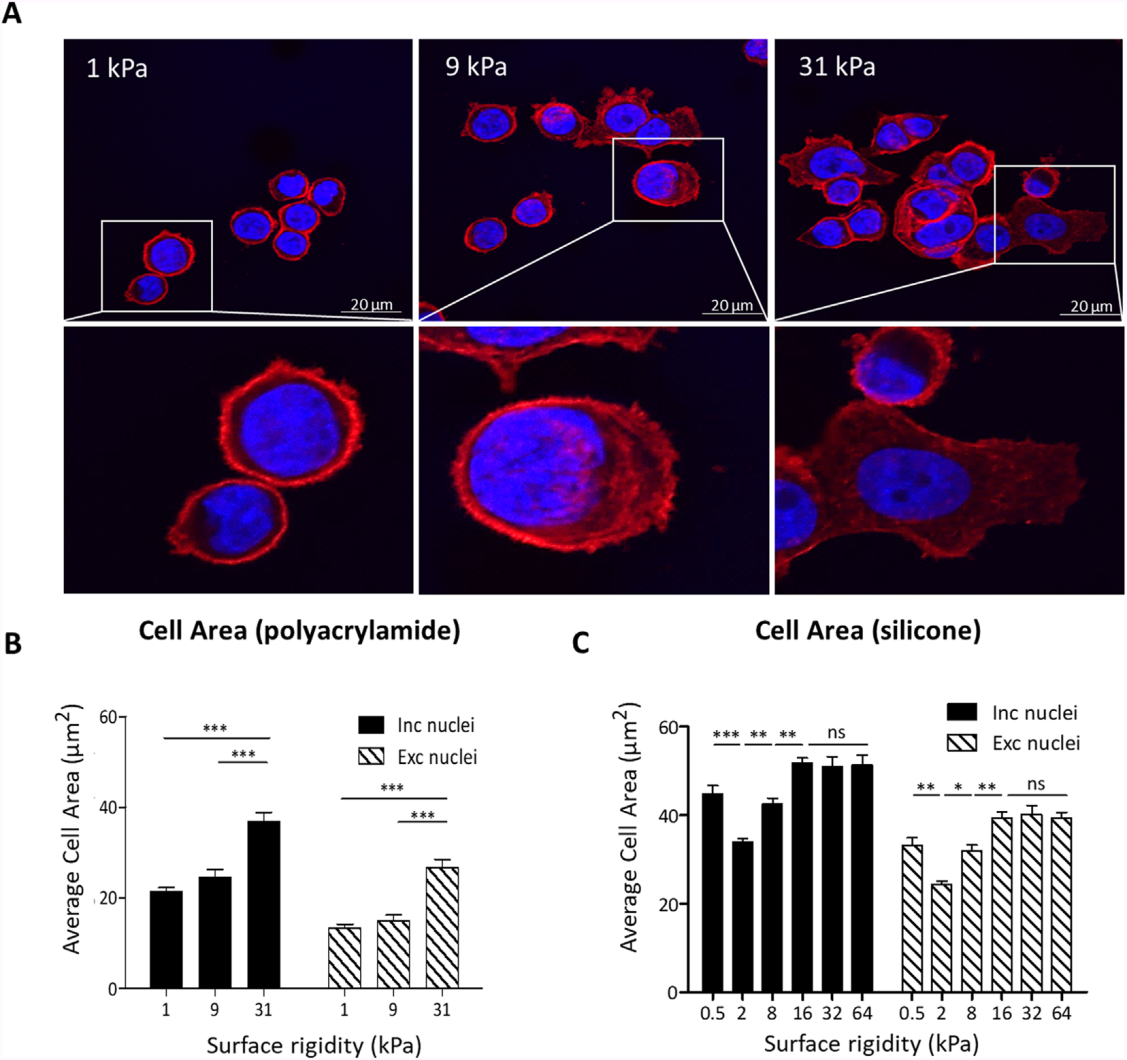
Higher cell spreading on stiffer surfaces. (a) Representative fluorescent images of AsPC-1 cells cultured for 12 h on fabricated polyacrylamide surfaces of varying stiffnesses, indicating their degree of spreading during microparticle incubation. Cells were stained with Phalloidin-iFluor 555 (red) and DAPI (blue). Scale bar: 20 *μ*m. The exposed cell surface available for particle absorption was evaluated using Photoshop for cells plated on (b) fabricated polyacrylamide and (c) commercial silicone substrates over a span of 12 h. The cells' surface area was calculated including and excluding the nuclei. n = 15. ^*^p < 0.05, ^**^p < 0.01, ^***^p < 0.001, and ns, not significant. Results are presented as the mean ± SEM. See also Figs. S3 and S4.

The exposed cell surface available for particle absorption is a critical steric factor that greatly influences the extent of particle uptake. Fluorescent images [Figs. 4(a) and S3] show that cells cultured on soft substrates displayed a round morphology, whereas those on intermediate and stiff substrates appeared to be more spread out. These findings are in agreement with previous studies.^33–35^

Moreover, the surface area of cells grown on intermediate substrates was smaller than those grown on stiff substrates. The exposed cell surface of cells plated on different substrates for 12 h was evaluated using Adobe Photoshop software [Figs. 4(b) and 4(c)]. Generally, we found a clear rigidity-dependent pattern of an increase in the exposed cell area with increasing surface rigidity, with cell area saturation for rigidities larger than 16 kPa. However, the exposed cell area of AsPC-1 cells grown on a 0.5 kPa surface deviated from this trend. This deviation, which, to the best of our knowledge, has not been previously observed, may result from the non-conformational behavior of the cells on very soft substrates, and it is probably not an indicative case. The cell area was measured both including and excluding the nuclei since particle uptake likely occurs predominantly outside of the nuclear region. In both cases, we observed very similar dependencies of the cells' surface area on surface rigidity, indicating that the issue of whether particles are or are not adsorbed on top of the nuclei does not qualitatively affect the dependence of uptake on the surface rigidity.

The average pixel-per-cell ratio is higher for silicone surfaces than for polyacrylamide surfaces of similar stiffness [Figs. 4(b) and 4(c)], implying that cells on silicone surfaces tend to have larger surface areas. This finding suggests that the material composition of surfaces influences cell spreading and attachment. To further investigate this, the adherence of AsPC-1 cells was evaluated after short incubation times (2 and 6 h after seeding), which showed that the cells adhere better to the silicone surfaces compared to the polyacrylamide ones (Fig. S6).

### Surface stiffness affects the uptake capacity of cells

**FIG. 5.**
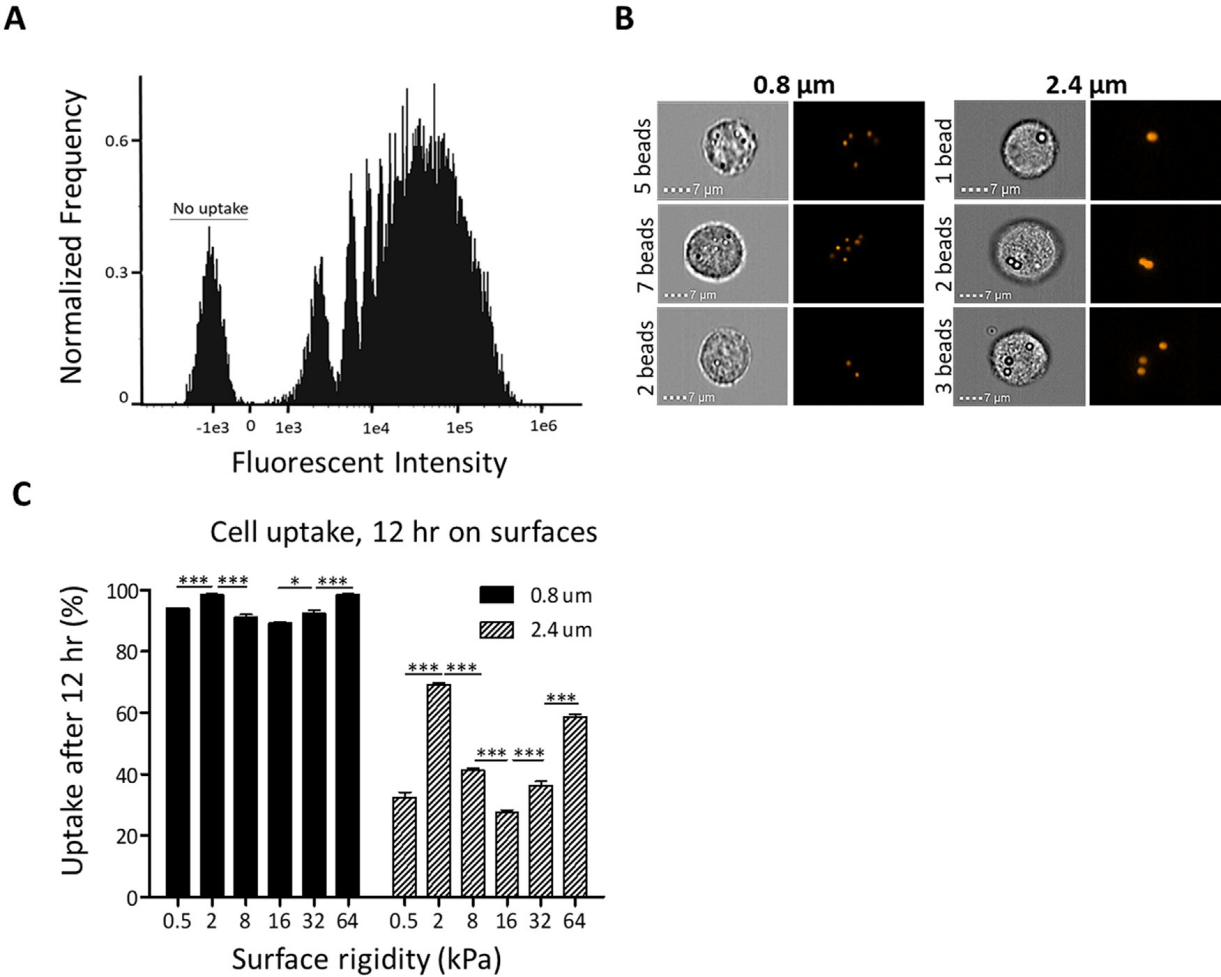
Surface stiffness affects the uptake capacity of AsPC-1 cells. (a) The bead uptake was evaluated using ImageStream and is displayed by fluorescent channel intensity histograms. The x-axis represents the integrated fluorescent intensity of the beads in the image. (b) Representative images of cells incubated with 0.8 and 2.4 *μ*m microbeads captured using ImageStream; left—brightfield images of the cells, right—fluorescent images of the beads. Scale bar: 7 *μ*m. (C) ImageStream uptake analysis was performed following the addition of 0.8 and 2.4 *μ*m fluorescent spherical beads, after 12 h of incubation with AsPC-1 cells seeded on commercial silicone surfaces of varying stiffnesses (0.5, 2, 8, 16, 32, and 64 kPa) (n = 3. ^*^p < 0.05, ^***^p < 0.001, and ns, not significant). The y-axis represents the percentage of the total cell population that internalized any number of beads. p-values presented in the graph show a significant non-monotonic dependence of the cells' uptake capacity on the surface rigidity. Results are presented as mean ± SEM. See also Figs. S5 and S7.

To investigate the relationship between surface stiffness and cellular uptake capacity, we measured the uptake of fluorescent polystyrene particles by AsPC-1 [Figs. 5 and S5(a), S5(b), S5 (d), S5(f)] and MDA-MB 231 [Figs. S5(c) and S5(e)] cells cultured on fabricated and commercial matrices [Figs. 5(c) and S5] of different rigidities.

We tested several incubation periods of AsPC-1 cells on surfaces of various stiffnesses and found that 12 h is an optimal time interval for cells to attach and spread on the gel surface while minimizing their penetration into the gel (Fig. S4). After the cells had been cultured for 12 or 96 h on surfaces of different rigidities, 0.8 or 2.4 *μ*m fluorescent polystyrene beads were added for a predetermined incubation time of 12 h. A non-monotonic dependence between the cell uptake capacity and the surface rigidity of commercial silicone substrates is observed in Fig. 5(c). The percentage of cells grown on surfaces with low rigidities, that internalized any number of beads (one or more), was lower compared with cells grown on surfaces of high rigidity. Whereas the percentage of cells cultured on surfaces with medium rigidity was the highest. When comparing the uptake capacity of 0.8 *μ*m beads with 2.4 *μ*m beads, both kinds of cells internalized larger beads less efficiently. Although the trend of the non-monotonic dependence of the cell uptake capacity on surface rigidity was preserved across both bead sizes, the difference in the uptake capacity of cells cultured on different surface rigidities became more apparent with larger beads. Similarly, to the commercial silicone substrates, we found that the fabricated polyacrylamide surfaces with increased surface rigidities did not induce a monotonic increase in the cell uptake capacity [Fig. S5(a)]; rather, there were alternating results.

Different cells have varied uptake kinetics for the same particles.^36^ When AsPC-1 cells were cultured with beads for 6 h, a maximum of 1.5% of the total cell population ingested one or more particles, as opposed to MDA-MB 231 cells, in which over 50% of the cell population ingested beads [Figs. S5(c) and S5(d)]. Incubation of MDA-MB 231 cells with beads for 12 h resulted in bead internalization by ∼95% of the cell population on all the different surface rigidities [Fig. S5(e)].

In most of the cases studied here, meandering uptake patterns were observed. The “wavelength” and “phase” of the wave-like dependence varied with cell type and particle size. Different cell lines responded differently to the mechanical and biochemical cues mediated by substrate rigidity. These parameters affect the cells' specific interactions with beads of different sizes. Among all the cases studied, the only one in which a monotonic pattern was found was in the MDA-MB 231 cells when incubated with 2.4 *μ*m beads [Fig. S5(c)]. This observation does not contradict a large wavelength situation, where non-monotonicity would be observed in a broader range of surface rigidities.

### Opposing trends controlling the dependence of the uptake capacity of cells on the surface rigidity

**FIG. 6.**
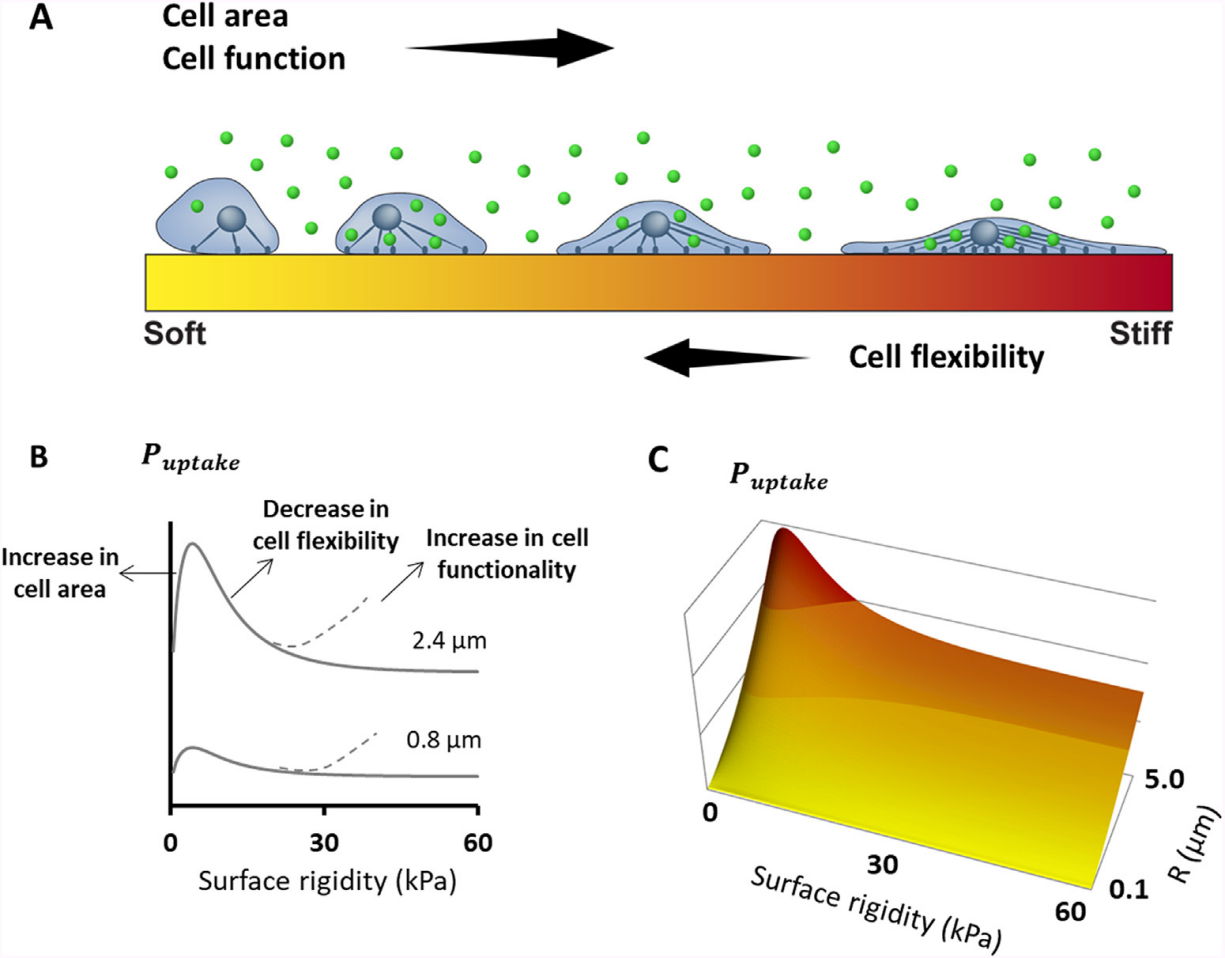
Opposing trends control the dependence of the uptake capacity of cells on surface rigidity. (a) Two major physical effects control uptake probability, assuming that stable micro-particle absorbance is a limiting step toward full particle engulfment. The first is the increase in the cell spreading area, which increases the number of particles that are in contact with each cell. The second is the elevation in cell stiffness, which increases the energetic penalty associated with the cell deformation needed for particle adhesion. (b) A physical model accounting for these two opposing effects is shown for 0.8 or 2.4 *μ*m beads in the solid curves of the plot. The apparent additional increase in uptake found in our experiments (denoted by the dashed lines) appears to be a nonphysical effect resulting from the elevated cell functionality on substrates of higher rigidity. This would cause an increase in the function describing the dependence of cellular uptake on surface rigidity, which may become dominant at any level of surface rigidity (in the example here it appears after the rigidity corresponding to peak uptake), contributing to the meandering behavior. (c) The non-monotonic dependence of particle uptake on the substrate rigidity, which is predicted from the physical arguments suggested here, reflects an initial increase followed by a decrease in uptake when increasing substrate rigidity. A surface plot also shows that the uptake is predicted to increase with the bead radius in this size scale, consistent with our previous findings on rigid substrates.^22^

Although the meandering uptake pattern may seem like wave dependence, there is no reason to assume that infinite periodicity is present in cell uptake behavior as a function of substrate stiffness. Instead, the non-monotonic dependence most likely results from a superposition of discrete opposing effects. To better understand the mechanical role in the meandering uptake pattern, we introduced a simple physical scenario that accounts for two conflicting trends, as illustrated in Fig. 6(a). The basic assumption, whose validity is examined below, and is by previous studies,^22,25^ is that phagocytosis begins as a slow thermodynamic process and that stable particle absorbance is a limiting step toward full particle penetration. Based on this assumption, understanding the physical interplay which governs static particle adhesion to the cell membrane provides valuable insights into the cell uptake dependence on surface rigidity. On the one hand, the cells are more spread out on stiffer substrates; thus, each cell has more surface area available to adsorb particles. However, cells are less deformable on more rigid substrates, and are therefore less likely to perform the shape deformation needed for contact with the micrometer-scale particles.^21,22^ For a simple intuitive understanding of the latter argument, it is assumed here that the uptake probability density function is linearly proportional to the initial cell-particle contact area, ɑ (ξ), where ξ is the substrate rigidity. The more elastic the cells are, the larger the particle wrapping by the cell membrane is during the initial absorbance. For micrometer-scale particles, this step is governed by the three-dimensional deformation of the cell bulk. It, therefore, depends on the cell's Young's modulus, E(ξ), which increases with the substrate rigidity.^37,38^ Thus, the more rigid the substrate, the smaller the ɑ (ξ); this tends to reduce the probability of proceeding into complete active particle internalization. This is in contrast to the increase in the cell spreading area, which increases the number of particles that are in contact with the cell and thus contributes to elevated uptake on more rigid substrates. Based on these arguments, the dependence of the uptake probability density on the substrate stiffness can be written as

$$P_{\mathrm{uptake}}^{}(\xi )\propto A(\xi )\cdot a(\xi ).$$

As we found in the fluorescent image analysis (Fig. 4), and in accordance with previous findings,^37,38^ the spreading area $A\left(\xi \right)$ of cells increases with ξ until saturated (excluding the divergent case of 0.5 kPa on commercial substrates). We use the approximate expression $A\left(\xi \right)\propto \tanh\left(0.2\xi \right)$ to describe the trend of the results in Fig. 4(c). We further assume that $E\left(\xi \right)\propto \tanh\left(0.05\xi \right)$, based on previous experimental findings.^38^ The cell-particle contact area during static absorbance was obtained using a thermodynamic model that we previously published,^22^

$$\alpha \left(\xi \right)=2\Pi R^{2}[1-\text{cos}\left(\Theta \left(\xi \right)\right)],$$

where the dependence on ξ is assumed to be governed by the increase in the elastic cell moduli, which controls the contact angle $\theta \left(\xi \right)=\sqrt[{3}]{\frac{0.75\pi }{E\left(\xi \right)R}}(\Delta \gamma -\frac{4\kappa }{R^{2}})$. Here, typical values were taken for the work of adhesion per unit area Δγ=0.002 κPa  μm and the membrane bending modulus $\kappa =1.9\cdot 10^{-5}\;{\kappa \mathrm{Pa}\;\mu m}^{3}$.^22,39^

Using this general scheme, $P_{\mathrm{uptake}\;}(\xi )$ is plotted in Fig. 6(b) for beads of 0.8 or 2.4 *μ*m, as used in our experiments. The opposing effects detailed above resulted in an initial increase in uptake, followed by a decline as a function of the substrate rigidity. The meandering patterns are found in Fig. 5(b) indicate additional contributions that may not be physical. The dashed lines in the plots of Fig. 6(b) indicate that the increase in uptake with large rigidities can result from the increased functionality of the cells (as found in Fig. 4), which is manifested in elevated uptake activity on the more rigid substrates.

### An inverse correlation exists between the levels of phosphorylated paxillin and the extent of microparticle uptake in AsPC-1 cells

**FIG. 7.**
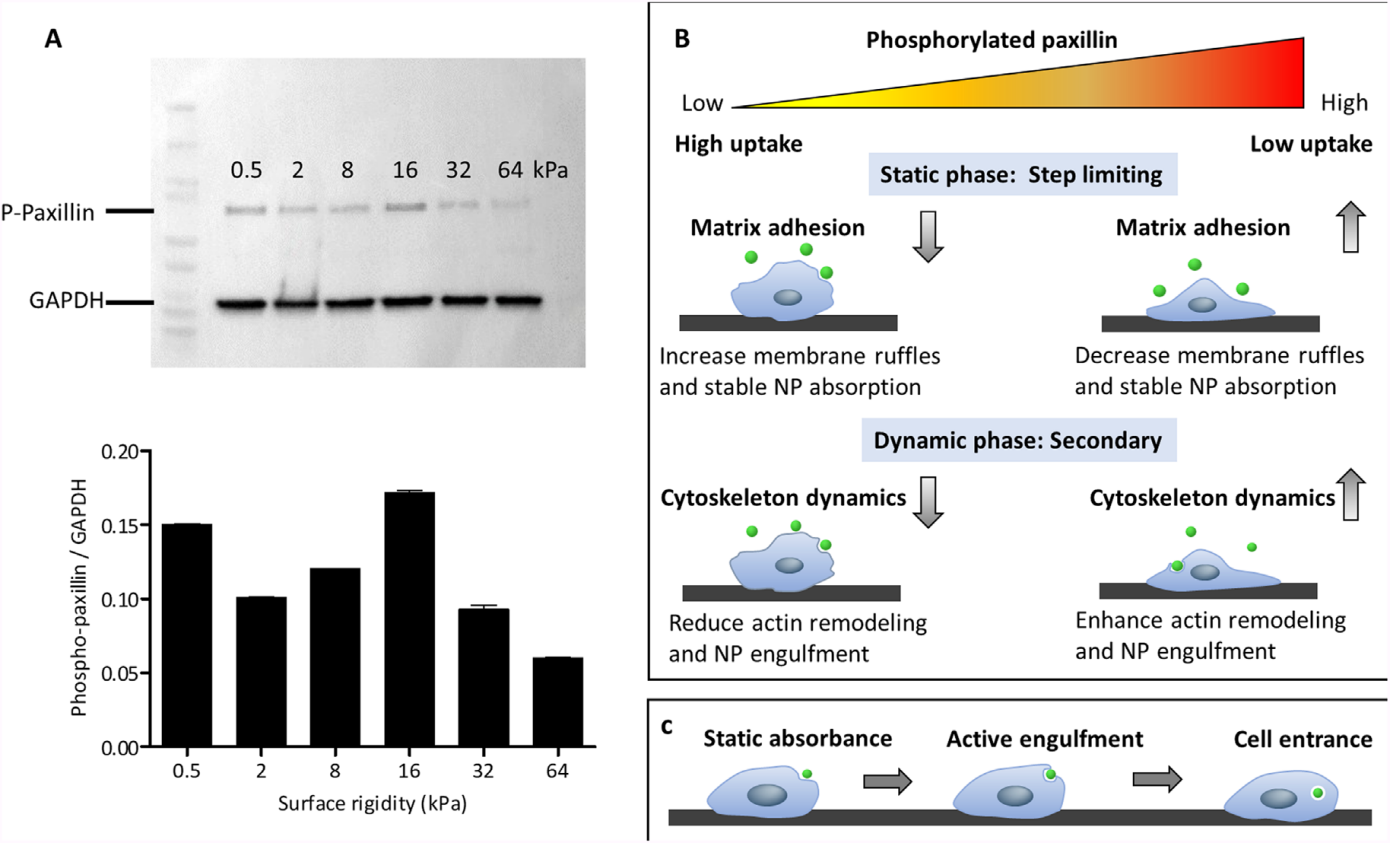
(a) Surface stiffness affects phosphorylated paxillin (Y118) expression in AsPC-1 cells. AsPC-1 cells were plated on commercial silicone surfaces of varying stiffnesses (0.5, 2, 8, 16, 32, and 64 kPa) for 12 h. Semi-quantitative western blot analysis to determine the expression of phosphorylated paxillin (68 kDa) is presented. The expression levels of glyceraldehyde-3-phosphate dehydrogenase (GAPDH) (37 kDa) were evaluated for normalization purposes. n = 2. (b) The inverse correlation between the levels of phosphorylated paxillin and the extent of micro-particle uptake (Fig. 5) indicates that stable particle adsorption to the outer cell membrane is a step limiting process that controls full entrance of the particle into the cell. We suggest that the major effect when phosphorylated paxillin is low is the release in cell tension, which allows for greater passive particle absorbance. The increase in cytoskeletal remodeling, which promotes active particle engulfment, was not the main factor in particle uptake; otherwise, there would be a direct correlation between uptake levels and phosphorylated paxillin expression. (c) We, therefore, suggest that the uptake of inert micro-particles by cells begins with thermodynamic adhesion. The second phase of active cytoskeletal remodeling is initiated if stable contact is formed, leading to major particle engulfment by the cell membrane. This process terminates when there is full particle entrance.

To gain mechanistic insights and further investigate the reliability of the assumption that stable particle adhesion to the cell membrane is required prior to complete cell penetration, we examined the expression of phosphorylated paxillin in AsPC-1 cells that were plated on substrates of varying rigidities [Fig. 7(a)]. Integrin binding to ECM promotes paxillin phosphorylation, activating numerous signaling cascades that have been shown to promote cell migration and adhesion dynamics.^40–42^ Inhibited levels of phosphorylated paxillin results in decreased cell motility and is correlated with weaker integrin-mediated matrix adhesion, resulting in an increased incidence of stress fiber breaks.^42,43^ The effect of phosphorylated paxillin on cell-matrix adhesion is expected to contribute mainly to the static phase of cell uptake, since reducing cell adhesiveness releases cell tension, manifesting in the formation of membrane ruffles.^44,45^ This thermal effect can reduce the need for the elastic alterations in cell morphology that are necessary for particle adhesion, as illustrated in Fig. 7(b). Notably, phosphorylated paxillin was also correlated with the remodeling of the actin cytoskeleton.^43,46^ Since actin dynamics are crucial for building phagocytic caps, and thus for the active phase of phagocytosis, low levels of phosphorylated paxillin are expected to reduce the probability of this phase occurring. Therefore, the dependence of the uptake on phosphorylated paxillin levels can indicate whether stable adhesion is a limiting step in the phagocytic process or whether the active phase is more important.

Strikingly, when comparing the expression levels of phosphorylated paxillin [Fig. 7(a)] with the uptake patterns found in Fig. 5, we found that they were inversely correlated, such that phosphorylated paxillin expression was high on substrates where the particle uptake was low and vice versa. This significant result indicates that static absorbance is the critical step necessary for active engulfment, since reduced phosphorylated paxillin decreases the energy barrier of the static absorption phase while downregulating the cytoskeletal dynamics required for active absorbance. This result constitutes a global finding regarding the stages of inert micro-particle uptake, which is relevant not only in the context of substrate rigidity but in cellular particle uptake in general,. The uptake stages are summarized in Fig. 7(c) and include initial thermodynamic adhesion, followed by active engulfment mediated by cytoskeleton remodeling and terminating with full cell penetrance.

## DISCUSSION

Cancer cell function is widely studied in the context of genetic and biomolecular cell profiles. It is well established that the tumor microenvironment dramatically affects cancer cell function due to the presence of various cytokines and secreted factors.^47–49^ However, beyond the biological molecular signals, the mechanical forces existing in a given tissue may have significant influence on cancer cell states. Indeed, hemodynamics and tissue mechanics expose cells to various physical forces that affect basic biological processes, such as cell division and differentiation.^50,51^

In a previous study, we established a clear link between cancer cell deformability and the capacity to engulf relatively large particles (sub-micron and micrometer) in a phagocytosis-like process that is facilitated by massive cell deformation. The cell's elasticity, rather than local changes in membrane fluidity, governs these significant shape distortions.^21^ This is particularly important since we found that microparticles can be used as mechanosensors for cell deformability, similar to AFM bead-attached tips. As a result of these discoveries, cancer cell phagocytosis-like behavior can serve an indicator for cancer cell function.

Although cancer cells do not have the specific phagocytic mechanisms of professional phagocytes, they still exhibit phagocytosis-like behavior and are highly efficient in internalizing large particles or foreign bodies. A striking example of this was demonstrated in the cannibalistic activity of melanoma cancer cells directed toward both cells and inert particles; interestingly, this activity resembles phagocytosis.^52^ Therefore, we focused our current work on the specific question of what happens to cancer cells when they attach to surfaces with various rigidities and whether this may affect their particle uptake patterns, which may correspond to mechanical-dependent cell function. A relevant scenario is whether cancer cells that leave their primary site, travel to a distant tissue, and attach to a new surface with a distinct rigidity behave differently or interact differently with external particles (synthetic or natural) than the cancer cells at the primary site. This may potentially be relevant for cells' biological activities as well as their response to therapies or uptake of imaging agents.

Another example of a clinically relevant scenario is physical changes that occur during tumor progression via changes in the microenvironment.^12,13,53^ In several cancers, such as pancreatic and breast cancer, desmoplasia occurs, where a dense and stiff ECM is formed.^54^ An increase in a tumor's microenvironment stiffness can result from the aberrant proliferation of the tumor cells, combined with deposition and remodeling of the ECM by the stromal environment.^55,56^ It was found that solid tumors are considerably stiffer than healthy tissues, correlating with cancer's progression and metastasis and compromising treatment efficacy.^57^ In this regard, it is possible for cancer cells to acquire different mechanical states within the same tissue and change their particle uptake pattern accordingly.

To examine the dependence of cancer cell uptake on surface rigidity, human pancreatic and breast cancer cells were incubated with inert and uncharged 0.8 or 2.4 *μ*m polystyrene beads. The substrate rigidity ranged from 0.5 to 64 kPa, representing the physiological conditions of most human tissues,^58^ and was found to affect the uptake capacity of the cells.

Interestingly, we found a non-monotonic dependence of particle uptake on substrate rigidity in a large range of experiments. There is no reason to believe that gel stiffness periodically affects the uptake pattern, as an increase in gel rigidity is not expected to cause a periodic effect on the gel properties. We, therefore, suggest that the meandering behavior results from a superposition of discrete physical and biological effects. Although some of the effects increase uptake with increasing substrate rigidity, others lead to the opposite effect. Thus, combining the overall factors results in a meandering uptake.

An important mechanistic insight was achieved by the inverse dependence that we found between the levels of phosphorylated paxillin [Fig. 6(a)] and particle uptake (Fig. 5). High levels of phosphorylated paxillin are expected to cause opposing effects. On the one hand, passive particle absorbance is reduced due to the release of cell tension, while on the other hand, active remodeling of the cytoskeleton is accelerated, which is required for particle wrapping by the cell membrane, whereas the first effect is expected to decrease micro-particle uptake, the second one is likely to enhance uptake. We could, therefore, use phosphorylated paxillin expression as an indication of the limiting step in the uptake processes. The inverse correlation between the phosphorylated paxillin levels and particle uptake indicates that the first (passive) stage of particle absorbance is crucial in determining the overall occurrence of uptake events. This led to a general conclusion about the stages involved in the uptake of inert microparticles by cells, as illustrated in Fig. 6(c). Stable absorbance initiates the process and is a necessary condition for the second stage, during which active remodeling of the cytoskeleton occurs. This enables large particle wrapping by the cell membrane until full cell penetrance is achieved.

The importance of the passive stage provides physical insight into mechanical aspects related to particle absorption. Figure 6(a) illustrates the fundamental physical factors that result in opposing trends. First, cells are more spread out on stiffer substrates, providing a larger surface available for interaction with particles. However, the cell spreading is not solely dependent on the surface rigidity. Figures 4(b) and 4(c) shows that cells tend to spread more on silicone surfaces compared with polyacrylamide surfaces of similar stiffness values. Additionally, we found that at short incubation times (2 and 6 h after seeding), AsPC-1 cells adhered more to silicone surfaces than polyacrylamide ones (Fig. S6), implying that surface biochemical properties affect and regulate cell adhesion behavior.^59^ This may be attributed to the varied local stiffness experienced by cells cultivated on various biomaterials due to different pore size and thickness of gels.^60^ Although the bulk moduli of polyacrylamide and silicone gels are quite similar, the combined effect of substrate thickness and microstructure may cause differences in cell adhesion behavior.^61^ Using image analysis, we also found an overall increase in the cell area on increasingly rigid substrates when excluding the nuclear regions, where uptake is less favorable (Fig. 4). This enhances the probability of phagocytosis on rigid substrates. On the other hand, cell spreading on rigid surfaces is associated with a non-linear increase in their elastic moduli, reducing the extent of particle uptake.^62^

Our findings show that cellular internalization of sub-micron and micrometer scale particles is size-dependent, and that the kinetics of uptake changes amongst cell types for the same type of particles (Figs. 5 and S5). The insertion of micrometer-scale particles into cells requires a mechanical distortion of the cells during the engulfment process. The decrease in the extent of uptake observed in Fig. S5(f) may be attributed to the partial or complete cell penetration into the gel surface (Fig. S4), reducing available cell area for particle interaction and limiting the cell's capacity to deform while wrapping the particle. In the initial (passive) phase of the interaction, elastic cell deformation occurs, which involves 2D membrane elasticity and 3D deformations of the cell bulk.^21^

It is reasonable to assume that the larger the cell-particle contact area during passive adhesion, the higher the probability of proceeding to active particle uptake, which involves mechano-biological pathways. Once a stable contact is established, particle uptake may occur through dynamic out-of-equilibrium processes involving active cytoskeletal remodeling along with eventual topological alterations of the cell membrane due to the detachment of the membrane-wrapped particle.^63^ The increased rigidity of the cells on stiffer substrates results in an elevated energetic penalty mainly associated with the three-dimensional cytoskeleton elasticity.^22^ Therefore, this factor is expected to reduce the passive engulfment and the subsequent dynamic uptake on more rigid substrates.

In addition to physical factors, the active processes during particle uptake are thought to correlate with the overall level of biological cell functionality. In an MTT assay, we found that the cells' metabolism was reduced by up to 30% on the softer substrates [Fig. 3(b)]. However, in a PI Annexin test, a high percentage of live cells was found on all substrates (Fig. S2). This indicates a reduction in the overall functionality of the cells when plated on flexible surfaces, supporting previous studies that showed that cells' molecular and biochemical activity depended on the surface rigidity.^64–66^ Thus, we suggest that this additional aspect contributed to the increase in uptake with elevated substrate stiffness, denoted by the dashed curves in Fig. 6(b).

In addition to the effects mentioned above, more aspects may be relevant in physiological conditions. One example is related to cell motility. Efficient surface exploration by cells can increase the fraction of particles that come into contact with them and consequently increase the uptake probability. It has previously been shown that within the range of rigidities tested here, surface exploration of cells is higher on less flexible surfaces.^38,67^ Thus, this factor presumably tends to increase the uptake with *ξ*. Another factor that may be relevant is that cells are expected to penetrate into softer surroundings.^68^ Here, short experimental durations were used to avoid this effect (Fig. S5). During these short incubation times, no differences were observed in the cell number between surfaces of varying stiffnesses (Fig. S7). Despite being less physiologically relevant than 3D models, monolayer cultures serve as a starting point for understanding various physiological processes and are considered straightforward models. Further investigation of uptake behavior could be conducted in 3D-tumor models, which can simulate physiological and morphological characteristics similar to the corresponding tumor *in vivo*.

## CONCLUSIONS

Our results indicate that cell states are largely affected by the mechanical surface to which the cells are attached and that the cell's ability to perform mechanically mediated uptake of large particles is the result of a combination of multiple factors, with passive adhesion being the limiting stage. Interactions between cancer cells and microparticles are relevant for many biologically and clinically relevant processes, including interactions with contrast agents (e.g., microbubbles), natural microparticles, and theopoetic carriers (e.g., drug-eluting embolic agents). Our findings could explain some of the heterogeneity in particle uptake in various organs. In addition, cancer cell phagocytic-like behavior was found to indicate their function, suggesting that cancer cells may acquire a more aggressive phenotype by changing their attachment to surfaces, e.g., the site on the body. From a mechanistic perspective, our findings suggest that inert microparticle uptake begins with passive particle adhesion to the cell membrane and continues with active cytoskeleton remodeling and particle wrapping. This insight is relevant not only for studying the mechanics of the environment, but also as a general feature of phagocytic-like uptake of inert particles. Further examination of these findings could provide additional mechanistic insight into the microparticle engulfment pathway.

## METHODS

Unless otherwise stated, reagents were purchased from Sigma-Aldrich. Fluorescently labeled purple polystyrene spherical particles 0.8 and 2.4 *μ*m in diameter were purchased from Spherotech (Lake Forest, Illinois, USA), Ex. 488 nm and Em. 545/60 nm.

### Cell culture

Human pancreatic adenocarcinoma cancer cell line AsPC-1 and breast adenocarcinoma cell line MDA-MB 231 were obtained from ATCC (VA, USA). AsPC-1 cells were maintained in RPMI medium, whereas MDA-MB 231 cells were maintained in Dulbecco's modified Eagle's medium (both from Life Technologies, MA, USA) supplemented with 10% fetal bovine serum and 1% antibiotics (10 000 *μ*g ml^−1^ streptomycin and 10 000 units ml^−1^ penicillin) at 37 °C with 5% CO_2_. AsPC-1 and MDA-MB 231 cells were mycoplasma-free (EZ-PCR Mycoplasma Test Kit Biological Industries, catalog number 2070020) and used for experiments until P15.

### Fabrication of polyacrylamide gel substrates

Polyacrylamide substrates were fabricated following an established protocol described by Fischer *et al.*^26^ The protocol was modified slightly in order to adapt it for two-dimensional (2D) cell culture conditions. Cover glasses (22 × 22 mm^2^) were activated using 0.5% 3-aminopropyltrimethoxysilane (Thermo Fisher Scientific, Heysham, UK) and 0.5% glutaraldehyde. Polyacrylamide gels of three different stiffnesses (1, 9, and 31 kPa) were formed on the activated surface by using different ratios of acrylamide and bisacrylamide (both from Bio-Rad, Israel). After preparation, gels were immediately used for experiments or stored at 4 °C with 100% humidity for up to 1 week. Gels were coated with collagen type I rat tail (Corning^®^ Life Sciences, USA). Hetero-bifunctional crosslinker Sulfo-SANPAH (Thermo Fisher Scientific, USA) was activated by UV light and used to coat the gel and allow the overlay of collagen on top. Gel substrates coated with collagen were left overnight at 4 °C to allow the collagen to bind to the gel. After incubation, the gel substrates were rinsed with phosphate-buffered saline (PBS) (Life Technologies, MA, USA). Prior to seeding cells, hydrogels were incubated at 37 °C for at least 24 h, allowing them to reach equilibrium swelling. To ensure that substrates of different rigidities were coated with equivalent amounts of ECM protein (collagen), the actual amount of collagen was quantified using a BCA protein assay kit (Thermo Fisher Scientific, USA) according to the manufacturer's instructions (Fig. S1). CytoSoft^®^ 6-well plates of varying stiffnesses (0.5, 2, 8, 16, 32, and 64 kPa) were purchased from Advanced BioMatrix, Carlsbad, California, USA. CytoSoft^®^ plates are coated with a 500 *μ*m-thick biocompatible silicone surface with a certified elastic modulus. Each lot of CytoSoft^®^ plates is independently tested by the manufacturer to ensure that its elastic modulus falls within quality control tolerances.^27,28^ According to the manufacturer's instructions, surfaces were coated with collagen type I rat tail prior to cell seeding. Briefly, 3.5 mg ml^−1^ collagen type I rat tail stock was diluted with warm PBS to create a working solution of 100 *μ*g ml^−1^. Collagen type I rat tail working solution was added to each well and incubated for 1 h at room temperature (RT).

#### 3D Printing of gel sample molds

All objects were designed using Autodesk AutoCad^®^ software, uploaded to the Asiga composer, and printed by an Asiga Max-X27 UV DLP-SL (digital light processing stereo lithography) printer (Sydney, Australia). The objects were printed using FotoTec^®^ DLP.A 380 nm resin (Dreve Otoplastik, Germany). After printing, the objects were gently removed from the build plate, rinsed and sonicated in isopropyl alcohol, dried, and then placed under a UV light to achieve complete resin curing.

### Substrate compression measurements

The mechanical properties of the polyacrylamide gels were measured in a parallel-plate compression configuration using a CellScale MicroTester (Canada). Results were analyzed using the SquisherJoy software program. The fabrication protocol for polyacrylamide gel substrates results in flat gels that are 25 *μ*m thick; however, the size range of samples that can be measured with the MicroTester is between 50 *μ*m and 2 mm. Therefore, polyacrylamide gel cubes were fabricated to fit the scale range of the MicroTester by casting them into 3D printed molds. After polymerization, the cube gels were removed from the molds using a 3D printed complementary template. Since the elastic modulus is an inherent feature of a material and does not vary with sample dimensions for the same formulation, we assume the elastic modulus of both surface and cube gel samples to be the same. All samples were tested in a PBS bath warmed to 37 °C. Prior to compression, the stainless-steel upper compression plate of the cantilever was lowered until it just contacted the top of the sample. Samples were compressed to 90% of their original size. Force-deformation curves were converted to stress-strain curves using the dimensions of the samples prior to their compression to calculate the polyacrylamide cube cross-sectional area. The Young's modulus was calculated by fitting the 10% strain data point with a linear regression line. A minimum of 10 distinct polyacrylamide cubes were examined for each gel stiffness to determine the average Young's modulus.

### Fluorescent microscopy cell imaging

For F-actin staining, AsPC-1 cells were cultured in CytoSoft^®^ 6-well plates or on fabricated polyacrylamide gels for 12 h. After 12 h, the cells were fixed with 4% paraformaldehyde (PFA) for 15 min, rinsed three times with 1× PBS, permeabilized with 0.1% Triton-X 100 (Thermo Fisher Scientific, Waltman, Massachusetts, USA) for 10 min, and then rinsed again 3 times with 1× PBS. Alexa Fluor^®^ 555 phalloidin Ex/Em 555/565 nm (Abcam, Cambridge, UK) was used according to the manufacturer's instructions. Nuclei were visualized using 4′,6-diamidino-2-phenylindole (DAPI) stain in a 1 *μ*g ml^−1^ solution (Thermo Fisher Scientific, Waltman, Massachusetts, USA). Images were obtained using an inverted fluorescent microscope (model IX73, Olympus Corporation, Japan) and a confocal microscope (Nikon's A1 MP multiphoton confocal microscope equipped with a 639-nm diode).

### Image analysis

The image analysis was performed using Photoshop software. In the images, cells appear in red (Alexa Fluor^®^ 555 phalloidin), nuclei in blue (DAPI), and the background in black. For the red and blue channels, two intensity-level thresholds were chosen to distinguish between pixels that indicate cells or nuclei, respectively. The percentages of cell and nuclei pixels were collected as the reciprocal of the integral of the histogram, from zero to the threshold level. This process was repeated for every image. The number of cells in an image was counted according to the blue nuclei DAPI staining.

### Imaging flow cytometer uptake studies

Cells were seeded on fabricated polyacrylamide gels and commercial gels of varying stiffnesses for 12 and 96 h. Fluorescently labeled polystyrene particles were diluted 1000-fold, added to the cells, and incubated for 6 (Fig. S5) or 12 (Fig. 5) h at 37 °C with 5% CO_2_. After incubation, the cells were washed with cold PBS, detached using trypsin A (Biological Industries, Catalog No. 03–050-1A), washed again, and filtered through a 40–50 *μ*m nylon mesh to remove cell debris. Cells were then centrifuged and suspended in a Fluorescence-activated cell sorting (FACS) buffer containing 1% Bovine Serum Albumin (BSA, VWR Chemicals, Solon, OH, USA) and 0.05% sodium azide n 1xPBS. Cell uptake capacity was quantified using ImageStream^X^ Mk II (Luminex, Austin, Texas, USA) and analyzed using IDEAS software. Focused cells were defined as cells with a gradient of root mean square (RMS)≥50. Single cells were identified as cells with a high aspect ratio and low area. Bead uptake was evaluated using fluorescent channel intensity histograms, where the y-axis represents the percentage of the total cell population that internalized any number of particles.

### Cell metabolic activity and apoptosis

AsPC-1 cells were seeded on fabricated polyacrylamide gels or on commercial gels at 300 000 cells per well with their complete culture medium. Cells were allowed to adhere and grow for 12 or 96 h. After incubation, MTT (3–(4,5-dimethylthiazol-2-yl)-2,5-diphenyl tetrazolium bromide) reagent was added into each well for metabolic activity detection and incubated at 37 °C with 5% CO_2_ for 4 h. Absorbance was measured at 540 nm using a plate reader (Wallac 1420 VICTOR plate-reader, Perkin-Elmer Life Sciences, USA). Absorbance values were transformed to metabolic activities, and percentage was calculated as the fraction of control cells that were cultured on standard 6-well plates (fixed as 100% metabolic activity).

Apoptosis was measured by staining cells with annexin V-APC and propidium iodide (PI) (Biolegends, San Diego, California, USA) according to the manufacturer's instructions after cells were grown on commercial gels for 12 h. After 12 h, cells were collected by centrifugation and washed with PBS. Ten thousand events per sample were recorded by FACS. Data analysis was performed using Flow-Jo software (BD Bioscience, USA).

#### Protein Analysis by Western Blotting

Western blotting analysis was performed to evaluate the phosphorylated paxillin Y118 expression levels in AsPC-1 cells grown on commercial surfaces of varying stiffnesses. For this purpose, cells were grown in their appropriate medium for 12 h, trypsinized, and lysed using the modified radioimmunoprecipitation assay buffer: 20 mM Tris-HCl (pH 7.5), 1% Nonidet-P40, 0.1% sodium dodecyl sulfate (SDS), 137 mM NaCl, 10% glycerol, complete protease inhibitor cocktail (S8820, Merck, Darmstadt, Germany), and Pierce Phosphatase Inhibitor Mini Tablets (Pierce, Thermo Scientific, MA, USA). Lysed cells were transferred to microcentrifuge tubes and sonicated four times for 5 s on ice. Insoluble cellular components were removed by centrifugation at 13 300 rpm for 10 min at 4 °C. Extracts (15 *μ*g protein) were mixed with 4× Laemmli sample buffer (Bio-Rad, CA, USA) containing 10% mercaptoethanol and boiled for 5 min. The protein content of the supernatant was determined according to the Bradford method using BSA as a standard. Proteins were separated by 4%–15% Mini-PROTEAN TGX Precast Protein Gel (200 mV, 30 min, Bio-Rad, CA, USA) and then transferred onto a polyvinylidene difluoride membrane (Millipore Corporation, Billerica, MA, USA) using a wet tank transfer system (100 V, 90 min, Bio-Rad, CA, USA). Membranes were blocked for 2 h with Tris-buffered saline 0.1% Tween 20 (TBST) containing 5% nonfat milk powder washed three times for 10 min in TBST, and incubated with 1:1000 diluted antibody for phosphorylated paxillin Y118 (#2541, Cell Signaling Technology, Massachusetts, USA) at 4 °C overnight in TBST containing 5% bovine serum albumin. The membranes were washed three times for 10 min in TBST and then labeled with a 1:3000 dilution of goat anti-rabbit secondary antibody conjugated to horseradish peroxidase (#7074, Cell Signaling Technology, Massachusetts, USA) in 5% nonfat dried milk for 1 h. The membranes were then washed again three times for 10 min in TBST, then 2 ml of the chemiluminescence substrate for HRP (Pierce, Thermo Scientific, MA, USA) was added for 2 min. The blots were imaged with a Bio-Rad gel imaging system and quantified with ImageJ software. GAPDH antibody (CST-2128S, Cell Signaling Technology, Massachusetts, USA) was also evaluated for normalization purposes.

### Cell adhesion assay

300 000 AsPC-1 cells were seeded per well on commercial silicone and fabricated polyacrylamide surfaces of varying stiffnesses. The seeding area of 22 × 22 mm coverslips with polyacrylamide gel of varying stiffnesses was 4.84 cm^2^. Each coverslip was placed in a 6-well plate with a seeding area of 9.5 cm^2^, which corresponds to the seeding area of commercial silicone surfaces. Cells were allowed to adhere to the surfaces of varying stiffnesses for 2 or 6 h. Hoechst was used to fluorescently label cells as directed by the manufacturer. The cells were then rinsed three times with PBS before being measured with a plate reader (Wallac 1420 VICTOR plate-reader, Perkin-Elmer Life Sciences, USA) in multiread-per-well mode. The same surface area was examined for fabricated and commercial surfaces.

### Statistical analysis

All experiments were performed and repeated at least three times, besides Western blotting, which was repeated twice. A parametric test and a two-tailed Student's *t*-test were used to calculate the significance of differences between two sets of results. An ANOVA test, followed by a Tukey post-hoc test, was used for multiple comparisons. Differences were considered statistically significant for *p* < 0.05.

## SUPPLEMENATRY MATERIAL

See the supplementary material for details: Figure S1. Quantification of collagen protein on the surface of polyacrylamide gels using the BCA assay. Figure S2. Annexin V-APC/PI assay for apoptosis evaluation. Figure S3. Cell spreading is higher on stiffer surfaces. Representative fluorescent images of AsPC-1 cells cultured for 12 h on commercial surfaces of varying stiffnesses. Figure S4. Cells penetrate softer hydrogels over time. Representative fluorescent images of AsPC-1 cells cultured on fabricated polyacrylamide surfaces of varying stiffnesses for 12 and 96 h. Figure S5. Surface stiffness affects AsPC-1 and MDA-MB 231 cells' uptake capacity. Figure S6. AsPC-1 cell adhesion behavior is influenced by surface biochemical properties. Figure S7. AsPC-1 cell counting after 12 h of incubation on fabricated polyacrylamide surfaces.

This project received funding from the European Research Council (ERC-StG) under the European Union's Horizon 2020 research and innovation program (Grant Agreement No. 0305260); The Israel Science Foundation (Grant Agreement No. 0394883); and the Israel Ministry of Science and Technology (MOST) (Grant Agreement No. 0394906). The funders had no role in study design, data collection, and interpretation, or the decision to submit the work for publication.

## Data Availability

All raw data are available upon request from the corresponding author.
